# Association between lowered endothelial function measured by peripheral arterial tonometry and cardio-metabolic risk factors – a cross-sectional study of Finnish municipal workers at risk of diabetes and cardiovascular disease

**DOI:** 10.1186/1471-2261-13-83

**Published:** 2013-10-11

**Authors:** Jussi Konttinen, Harri Lindholm, Juha Sinisalo, Eeva Kuosma, Janne Halonen, Leila Hopsu, Jukka Uitti

**Affiliations:** 1 Finnish Institute of Occupational Health, Topeliuksenkatu 41 a, A, FI-00250 Helsinki, Finland; 2 Helsinki University Central Hospital, Helsinki, Finland; 3 Finnish Institute of Occupational Health, Uimalankatu 10, FI-33101 Tampere, Finland

**Keywords:** Cardiovascular risk, Endothelial dysfunction, Peripheral arterial tonometry, Occupational health care

## Abstract

**Background:**

The aim of this cross-sectional study was to determine the association between lowered endothelial function measured by peripheral arterial tonometry (PAT) and cardio-metabolic risk factors. The study population consisted of Finnish municipal workers who were at risk of diabetes or cardiovascular disease and who had expressed a need to change their health behaviour.

**Methods:**

A total of 312 middle-aged municipal workers underwent a physical medical examination and anthropometry measurements. Levels of total cholesterol, HDL cholesterol, triglycerides, fasting glucose, glycated haemoglobin, and high sensitivity C-reactive protein were taken from the blood samples. PAT measured the increase in digital pulse volume amplitude during reactive hyperemia, and the index of endothelial function, F-RHI, was defined as the ratio of post-deflation amplitude to baseline amplitude.

**Results:**

In the linear regression model, male sex was associated with lower F-RHI. In sex-adjusted linear regression models, each of the variables; waist circumference, fasting glucose, glycated hemoglobin, triglycerides, body fat percentage, body mass index, current smoking, and impaired fasting glucose or diabetes were separately associated with lower F-RHI, and HDL cholesterol and resting heart rate were associated with higher F-RHI.

HDL cholesterol, sex, body mass index, and current smoking entered a stepwise multivariable regression model, in which HDL cholesterol was associated with higher F-RHI, and smoking, male sex and body mass index were associated with lower F-RHI. This model explains 28.3% of the variability in F-RHI.

**Conclusions:**

F-RHI is associated with several cardio-metabolic risk factors; low level of HDL cholesterol, male sex, overweight and smoking being the most important predictors of a lowered endothelial function. A large part of variation in F-RHI remains accounted for by unknown factors.

## Background

The assessment of traditional risk factors is a basic element in the primary prevention of cardiovascular disease (CVD). New markers of increased risk or pre-symptomatic disease are being studied in order to refine risk assessment and to target the treatment of those at highest risk [1]. The vascular endothelium plays a central role in the pathogenesis of CVD [2]. The measurements of endothelial function are promising indicators of vascular health [2].

The term “endothelial dysfunction” refers to a broad alteration in endothelial phenotype and capacity to maintain vascular homeostasis [2,3]. Endothelial dysfunction contributes to the initiation and progression of atherosclerotic disease and is an individual cardiovascular risk factor with prognostic value [2,3]. Many interventions that reverse endothelial dysfunction also reduce cardiovascular risk, and one potential use for the measurements of endothelial function is the evaluation of interventions to reduce CVD risk [2].

The most commonly used method to assess endothelial function is to measure endothelium-dependent vasodilatation by using stimuli that increase the production of endothelium-derived nitric oxide [2]. The flow-mediated dilatation in the brachial artery can be measured by vascular ultrasound [4]. Peripheral arterial tonometry (PAT) is a novel method for evaluating endothelial function. It has been used in ambulatory settings and in large scale trials [5,6]. PAT measures the increase in digital pulse volume amplitude during reactive hyperemia in relation to baseline. This response is reproducible, and has shown to be mainly dependent on nitric oxide [7,8]. The response is attenuated and the PAT score is lower in patients with coronary endothelial dysfunction [9]. There is also a significant relationship between PAT measures and brachial artery ultrasound measures [10]. Endothelial dysfunction measured by PAT is associated with multiple cardiovascular risk factors [5,10-13]. The changes in PAT-related endothelial function are reversible through effective interventions [14-16], and may be useful for the identification of patients at risk of cardiovascular adverse events [17].

This cross-sectional study aims to investigate the association between the index of endothelial function measured by PAT, F-RHI (Framingham reactive hyperemia index), and heterogenous cardio-metabolic risk factors in a population of asymptomatic municipal workers.

## Methods

### Study design

This study is part of the larger Nuadu interventional study, which aimed to create new methods of early recognition of people at health risks and a multi-factorial lifestyle intervention suitable for occupational health care [18]. Study subjects were selected from among municipal workers (n = ~10900) in the City of Espoo, based on a health questionnaire. A total of 4134 (38%) replied. The questionnaire included items on physical activity, sleep, smoking, drinking, nutrition, physical and mental stress and strain, perceived health, and work ability. The inclusion criteria were willingness to participate in the intervention, age between 30–55 years, and a subjectively estimated work ability of 7–9 on a scale of 0–10, 10 being lifetime best [19]. Another important criterion was the willingness to change health behaviour in one or more of the following target areas within the next six months: weight control, physical activity, alcohol consumption, eating habits, smoking or sleeping habits. Subjects also had to have an increased diabetes risk on the basis of a questionnaire (12–20 points in the Diabetes risk test) [20] or at least two of the following risk factors: body mass index (BMI) 27–34.9, low physical activity level [21], sleep deprivation (difference between self-estimated need of sleep and actual sleeping time greater than two hours), risky alcohol consumption based on AUDIT-C [22,23], smoking, not eating vegetables daily and/or not eating during the working day. Pregnant women were excluded from the study. Among the respondents, 783 (19%) employees fulfilled the inclusion criteria. Of these, 352 were randomly selected for the intervention study. Seventeen people who had a BMI of 35 or higher were excluded.

Participants of the intervention study answered the basic Nuadu intervention questionnaire, underwent the baseline measurements of the intervention, and received personal feedback on their situation. Two randomised groups participated in health behaviour interventions and one served as the control. The questionnaires and measurements were repeated one year later. The results presented here are those of the baseline measurements. Due to this, two intervention groups and a control group were pooled in this article.

All subjects gave informed consent and the study protocol was approved by the Coordinating Ethics Committee of the Hospital District of Helsinki and Uusimaa.

### Physiological measurements

Every participant underwent a medical physical examination before the physiological measurements. We measured height, weight and waist circumference (WC). Body composition was analysed by eight-polar bioelectrical impedance (BIA) (Body 3.0, Biospace Company Ltd, Seoul, South Korea). The blood samples were analysed according to the standard methods of the laboratory of Helsinki University Central Hospital (Accredited laboratory, http://www.huslab.fi). They included total cholesterol, HDL cholesterol, triglycerides, fasting glucose, glycated hemoglobin (HbA1c) and high sensitivity C-reactive protein (hs-CRP). LDL cholesterol was estimated using the Friedewald equation [24].

### Coding of dichotomous risk factors

Dichotomous risk factors were coded as 0 = absence of risk factor, and 1 = presence of risk factor. The criteria for coding the presence of risk factors were as follows: 1. Current smoking based on the intervention questionnaire; regular smoker, or casual smoker who was a regular smoker in the earlier health questionnaire; 2. Family history of cardiovascular disease: myocardial infarction, coronary revascularization, or sudden death of father or other male first degree relative before 55 years of age, or mother or other female first degree relative before 65 years of age; 3. Hypertension treatment or lipid lowering treatment: medication used regularly or often; 4. Impaired fasting glucose or diabetes: in laboratory measurements a fasting glucose of ≥ 6.1 mmol/l; 5. Hypertension: systolic blood pressure of ≥ 140 mm Hg and/or diastolic blood pressure of ≥ 90 mm Hg; 6. Dyslipidemia: an LDL cholesterol of < 3 mmol/l, triglycerides of > 2, an HDL cholesterol of < 1 mmol/l and/or a total to HDL ratio of > 4. In regression models, male sex was also considered a risk factor.

### Measurements of endothelial function

We measured the endothelial function of 312 participants. Digital pulse volume amplitude was measured and analysed using the PAT method (Endo-PAT2000, Software version 3.0.3, Itamar Medical Ltd, Caesarea, Israel). The principle of PAT has been described earlier [10]. During the assessment, the participant was in a supine position in a comfortable, quiet, and thermoneutral environment, with the lights dimmed.

Pneumatic probes were placed on the index finger of each hand, and a blood pressure cuff was placed on the left upper arm. The left hand underwent reactive hyperemia testing and the contralateral hand served as the control. The participants were allowed to lie down for a couple of minutes before the recording of the pulsatile volume changes began. After five minutes of baseline measurement, the blood pressure cuff was rapidly inflated to 200 mm Hg or 60 mm Hg above systolic blood pressure, whichever was higher. Occlusion lasted for exactly five minutes, after which the blood pressure cuff was deflated to induce reactive hyperemia. We continued recording for five more minutes.

The PAT data was analysed by a computerised operator independent algorithm with Endo-PAT2000 software. The manufacturer’s basic index of vascular reactivity, RHI, is defined as the ratio of postdeflation pulse amplitude to the baseline pulse amplitude. The average amplitude of a one-minute period of PAT signal, starting one minute after cuff deflation, is divided by the average amplitude of the 2.5-minute period of PAT baseline signal before cuff inflation. This ratio is normalized to the corresponding ratio from the control arm to compensate for potential systemic changes in the amplitude. The automatic calculation of RHI has an imbedded correction factor to deal with the strong negative correlation between the baseline pulse amplitude and the relative response. Baseline pulse amplitude itself correlates with many cardiovascular risk factors, and the correction factor may actually hide some relevant information. Thus in this study, in addition to RHI, we calculated and used an index without the correlation factor. F-RHI was defined as F-RHI = ln(RHo/RHc), where RHo and RHc are the mean pulse amplitudes of the period 1.5–2 minutes after cuff deflation divided by mean baseline amplitudes in the occluded arm and control arm, respectively [11]. F-RHI is more highly associated with cardiovascular risk factors and has also shown to be more reproducible than RHI in adolescents [7].

### Statistical methods

A linear regression model was used to determine the association of F-RHI with sex. We used twenty separate sex-adjusted linear regression models to find the association of F-RHI with each of the following cardiovascular and metabolic risk factors: age, BMI, WC, body fat percentage assessed by BIA, total cholesterol, HDL cholesterol, LDL cholesterol, triglycerides, fasting glucose, HbA1c, hs-CRP, systolic and diastolic blood pressure, resting heart rate, rate-pressure product, hypertension treatment, lipid lowering treatment, impaired fasting glucose or diabetes, family history of CVD, and current smoking.

The normality of the variables was tested using the Kolmogorov-Smirnov test. CRP values were log-transformed for the regression models to obtain normal distribution.

We selected the variables that were allowed to enter the multivariable regression model in advance to avoid co-linearity. As regards possible co-linear variables, we selected the potential covariate based on current knowledge on the importance of the risk factors and the reliability of the method used to measure it. In the multivariable regression model we used a stepwise method for variable selection. The criteria for entering or keeping the variable in the model were p < 0.05 and p < 0.1, respectively. All analyses were performed using SPSS 15.0 for Windows (SPSS Inc., Chicago, IL, USA).

## Results

### Subject characteristics

Subject characteristics are presented in Tables 1 and 2.

**Table 1:** Subject characteristics tabulated by sex

**Cardiovascular and metabolic risk factors**	**Women (n = 254)**	**Men (n = 58)**	**p**
Age (y)	44.6 ± 6.7	43.5 ± 7.6	0.278
Body mass index (kg/m^2^)	27.2 ± 4.1	27.6 ± 3.6	0.406
Waist circumference (cm)	90.4 ± 11.3	100.0 ± 10.1	<0.0001
Body fat percentage (%)	31.6 ± 6.6	22.2 ± 6.0	<0.0001
Total cholesterol (mmol/l)	4.7 ± 0.8	5.0 ± 1.0	0.039
HDL cholesterol (mmol/l)	1.6 ± 0.4	1.3 ± 0.3	<0.0001
LDL cholesterol (mmol/l)	2.6 ± 0.7	3.1 ± 0.9	<0.001
Triglycerides (mmol/l)	1.0 ± 0.6	1.4 ± 0.7	<0.001
Fasting glucose (mmol/l)	5.4 ± 0.8	5.5 ± 0.6	0.226
Glycated hemoglobin (%)	5.5 ± 0.4	5.6 ± 0.3	0.264
High sensitivity C-reactive protein (mg/l)	2.11 ± 3.97	1.96 ± 3.08	0.912
Systolic blood pressure (mm Hg)	121 ± 13	125 ± 14	0.015
Diastolic blood pressure (mm Hg)	79 ± 8	81 ± 9	0.041
Resting heart rate (bpm)	69 ± 10	69 ± 11	0.619
Rate-pressure product	84 ± 16	86 ± 18	0.327
Framingham Reactive Hyperemia Index	0.676 ± 0.401	0.325 ± 0.293	<0.0001
Reactive Hyperemia Index	2.272 ± 0.617	1.903 ± 0.403	<0.0001

Values are mean ± SD. P-value for differences of means between sexes.

**Table 2:** Risk factors and medication tabulated by sex

**Cardiovascular and metabolic risk factors**	**Women (n = 254)**	**Men (n = 58)**	**p**
Current smoking	20.9	25.9	0.406
Body mass index > 25	65.4	81.0	0.021
Family history of cardiovascular disease	28.0	20.7	0.259
Impaired fasting glucose or diabetes	9.4	12.5	0.491
Hypertension treatment	11.0	5.2	0.179
Lipid lowering treatment	3.1	6.9	0.189
Hypertension	13.4	24.1	0.041
Dyslipidemia	31.1	67.9	<0.0001

Values are percentages, p-value for chi-square test.

### Associations of F-RHI with cardiometabolic risk factors

Male sex was associated with lower F-RHI in the separate linear regression model.

**Figure 1:**
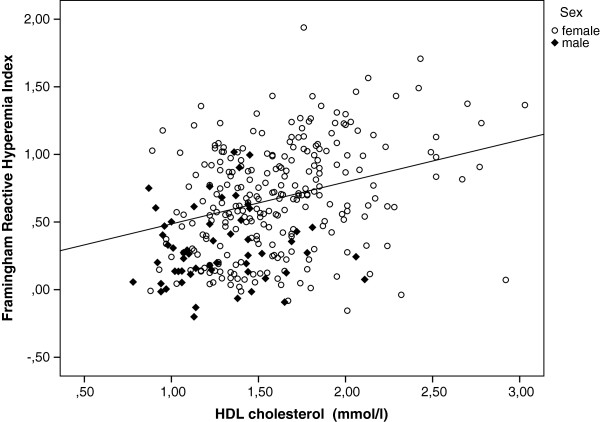
**F-RHI and HDL cholesterol.** F-RHI and HDL cholesterol values. Plot showing data points labelled separately for men and women and fitted regression line adjusted for sex.

Separate sex-adjusted linear regression models were used to find the association of F-RHI with each of the other risk factors. In these models WC, fasting glucose, glycated hemoglobin, triglycerides, body fat percentage, BMI, current smoking, and impaired fasting glucose or diabetes were inversely associated with F-RHI. The inverse relationship between F-RHI and C-reactive protein was nearly significant (p = 0.085). HDL cholesterol (Figure 1) and resting heart rate were positively associated with F-RHI. No significant relationships were found between F-RHI and age, systolic or diastolic blood pressure, pulse pressure, rate-pressure product, total cholesterol, LDL cholesterol, lipid lowering treatment, hypertension treatment or family history of CVD. Table 3 shows the results.

**Table 3:** Sex-adjusted associations of risk factors with F-RHI

**Cardiovascular and metabolic risk factors**	**Beta(se)**	**p**	**Partial correlation**
Sex	-0.352(0.056)	<0.0001	-0.337
Age	-0.004(0.003)	0.185	-0.075
Body mass index	-0.29(0.005)	<0.0001	-0.304
Waist circumference	-0.10(0.002)	<0.0001	-0.280
Body fat percentage	-0.011(0.003)	<0.001	-0.194
Total cholesterol	0.011(0.025)	0.656	0.025
HDL cholesterol	0.310(0.056)	<0.0001	0.302
LDL cholesterol	-0.025(0.029)	0.390	-0.049
Triglycerides	-0.123(0.035)	<0.001	-0.197
Fasting glucose	-0.100(0.029)	<0.001	-0.193
Glycated hemoglobin	-0.147(0.061)	0.017	-0.136
Log-transformed high sensitivity C-reactive protein	-0.024(0.014)	0.085	-0.100
Systolic blood pressure	0.00(0.002)	0.968	0.002
Diastolic blood pressure	-0.001(0.003)	0.825	-0.013
Resting heart rate	0.005(0.002)	0.023	0.130
Rate-pressure product	0.002(0.001)	0.084	0.099
Hypertension treatment	-0.073(0.073)	0.316	-0.057
Lipid lowering treatment	-0.031(0.113)	0.786	-0.015
Impaired fasting glucose or diabetes	-0.140(0.070)	0.047	0.113
Family history of cardiovascular disease	0.004(0.049)	0.940	0.004
Smoking	-0.152(0.052)	0.004	-0.164

Slope (standard error), p-value, partial correlation coefficient.

For multivariable analysis, we selected the variables that were allowed to enter the model in advance. Sex, BMI, current smoking, fasting glucose, triglycerides, HDL cholesterol, resting heart rate and C-reactive protein were significantly or nearly significantly associated with F-RHI in separate sex-adjusted models and were allowed to enter the multivariable model. In addition, age, LDL cholesterol, blood pressure and family history of CVD were allowed to enter the model as strong risk factors for CVD or diabetes. The variables selected by the stepwise procedure for the final model from those listed above were HDL cholesterol, sex, BMI and current smoking. This model explains 28.3% of the variability in F-RHI. Table 4 shows the results.

**Table 4:** Final stepwise multivariable regression model

**Cardiovascular and metabolic risk factors**	**Beta(se)**	**p**	**Partial correlation**
HDL cholesterol	0.240 (0.060)	<0.0001	0.229
Sex	-0.264 (0.057)	<0.00001	-0.249
Body mass index	-0.026 (0.006)	<0.00001	-0.248
Smoking	-0.154 (0.050)	0.0022	-0.157
^1^Model R^2^	0.283		

Slope (standard error), p-value and partial correlation and ^1^square of multiple correlation coefficient for model.

### Additional analysis

To further estimate if the risk factors play differently in men and women we made separate multivariable models for the two sexes. The variables that were selected for the final model for women were HDL cholesterol, BMI and smoking. The model explains 20.0% of the variability in F-RHI. As regards men, only BMI entered the model, which explains 13.8% of the variability. Tables 5 and 6 show the results.

**Table 5:** Stepwise multivariable regression model for women

**Cardiovascular and metabolic risk factors**	**Beta(se)**	**p**	**Partial correlation**
HDL cholesterol	0.282 (0.064)	<0.0001	0.273
Body mass index	-0.024 (0.006)	<0.001	-0.244
Smoking	-0.157 (0.057)	0.007	-0.161
^1^Model R^2^	0.200		

Slope (standard error), p-value, partial correlation and ^1^square of multiple correlation coefficient for model.

**Table 6:** Stepwise multivariable regression model for men

**Cardiovascular and metabolic risk factors**	**Beta(se)**	**p**	**Partial correlation**
Body mass index	-0.031 (0.011)	<0.007	-0.371
^1^Model R^2^	0.138		

Slope (standard error), p-value, partial correlation and ^1^square of multiple correlation coefficient for model.

## Discussion

Several cardiovascular and metabolic risk factors were associated with lowered endothelial function, measured by the PAT method, among those at risk of diabetes and cardiovascular disease. HDL cholesterol, current smoking, sex and body mass index explain more than a fourth of the variability in F-RHI.

As in previous studies, lower F-RHI was strongly associated with male sex in the separate linear regression model and the multivariable model [11,13]. Since vascular function and development of cardiovascular disease differ between the sexes, it would be useful to study men and women separately instead of using sex as a covariate [25]. We performed additional analyses to find possible sex differences in associations between F-RHI and risk factors, but because of the low proportion of male subjects, we were unable to build a reliable model for men alone.

Lower F-RHI was strongly associated with obesity. The strongest single association we found was between F-RHI and BMI. Similar results can be obtained with body fat percentage or WC. In separate sex-adjusted models, all the inter-correlated measures of obesity; body mass index, waist circumference and body fat percentage measured by bioelectrical impedance analysis, were related to lower F-RHI. Of these, BMI was chosen as a candidate for the multivariable model and thus entered the model. This observation was in accordance with earlier studies [11,13].

Disturbed lipid profile associated with lower F-RHI. In separate sex-adjusted models, high HDL cholesterol was associated with higher F-RHI, and high triglycerides associated with lower F-RHI. HDL cholesterol entered the multivariable model, and was one of the strongest predictors of F-RHI. However, there were no associations between F-RHI and total cholesterol, LDL cholesterol, or lipid-lowering treatment. The lipid values in the study population were better than those of the Finnish working age population in general [26]. One study with dominantly male subjects found a significant association between LDL cholesterol and lower PAT score, but none with HDL cholesterol, triglycerides or hyperlipidemia treatment [13]. Hamburg et al. found significant associations between lower F-RHI and total/HDL cholesterol, triglycerides and lipid-lowering treatment [11].

The findings concerning diabetes, fasting glucose and current smoking also support earlier studies [11].

Inflammation plays an important role in the pathogenesis of atherosclerosis [27]. We found no significant association between a marker of systemic inflammation, hs-CRP and F-RHI, although there was a modest non-significant association between higher log-transformed hs-CRP and lower F-RHI in the sex-adjusted model.

Age was not associated with F-RHI in the sex-adjusted model or in the multivariable analysis. It is possible that the range and distribution of age in our study population was not sufficient to show a possible relation. Earlier studies have shown a small but paradoxically positive association between advancing age and higher F-RHI [11], or no association at all between age and F-RHI [13].

Associations between F-RHI and blood pressure or resting heart rate were modest and somewhat counterintuitive. We found no association between F-RHI and systolic or diastolic blood pressure. Higher resting heart rate was paradoxically associated with higher F-RHI in the sex-adjusted model, although the beta coefficient was very small. Truschel et al. [13] found a significant but counterintuitive positive association between F-RHI and systolic blood pressure, but no relationship with resting heart rate [13]. Hamburg et al., conversely found an association between lower F-RHI and higher heart rate [11].

Blood pressure values in our study population were quite low compared to the Finnish working-age population in general [26]. More hypertensive data might possibly provide better information on the association.

Although the result of the PAT measurement, F-RHI, is mainly nitric oxide dependent, it is likely to be a combination of macro- and micro-vascular reactivity and affected by skin blood flow changes [28]. It is possible that the hyperemic response in the fingertip microvessels differs from other vascular beds [11]. This study confirms some of the associations found earlier between F-RHI and certain risk factors. Many of the established cardio-metabolic risk factors are associated with lower F-RHI as expected, but in this study the relationships with age, blood pressure and heart rate in particular are not parallel with the results of other studies, and need further investigation.

The study’s strengths lie in the relatively large sample size and a good coverage of municipal workers at intermediate cardio-metabolic risk. We were also able to determine a comprehensive set of risk factors and study their associations with a novel measure of endothelial function.

One limitation of this analysis is its cross-sectional design. In addition, the number of male subjects was low, because the proportion of females among Finnish municipal workers is commonly significantly higher. Measurements of vascular reactivity were not carried out in a fasting state due to the study design, although the subjects were instructed to eat only lightly before the measurements. A study using an ultrasound technique for measuring endothelial function suggests that strict requirements for fasting conditions may be unnecessary [29].

The menstrual cycle phase was not controlled for. The women ranged from peri- to post-menopausal, but information regarding hormonal status or the use of menopausal hormones was not collected. In addition to sex, hormonal status contributes to and modulates the development of cardiovascular disease and should be taken in to account in future study design [25].

The study sample consisted of Caucasian people of European origin, thus the findings may not apply outside this population.

## Conclusions

In conclusion, the index of endothelial function, F-RHI, measured by PAT is associated with several cardio-metabolic risk factors; low HDL cholesterol level, male sex, overweight and current smoking being the most important predictors of a lowered F-RHI. A large part of the variation in F-RHI is still explained by other factors, and relationships between F-RHI and age or hemodynamic indices in particular need further investigation.

## Abbreviations

CVD: Cardiovascular disease; PAT: Peripheral arterial tonometry; F-RHI: Framingham reactive hyperemia index, the PAT score; BMI: Body mass index; WC: Waist circumference; BIA: Bioelectrical impedance analysis; hs-CRP: High sensitivity C-reactive protein; HbA1c: Glycated hemoglobin.

## Competing interests

The authors declare that they have no competing interests.

## Authors’ contributions

JK participated in the design of the study; in the acquisition, analysis and interpretation of data and the drafting of the manuscript. HL participated in the design of the study, in the acquisition and interpretation of data, and helped draft the manuscript. JS participated in the design of the study, interpretation of data, and the drafting of the manuscript. EK participated in the design of the study and the design of statistical methods and helped draft the manuscript. JH participated in the acquisition of data and helped draft the manuscript. LH and JU participated in the design of the study and the drafting of the manuscript. All authors read and approved the final manuscript.

## Pre-publication history

The pre-publication history for this paper can be accessed here:

http://www.biomedcentral.com/1471-2261/13/83/prepub

## References

[B1] Greenland P Smith SC Jr Grundy SM Improving coronary heart disease risk assessment in asymptomatic people: role of traditional risk factors and noninvasive cardiovascular tests Circulation 2001 104 15 1863 1867 10.1161/hc4201.097189 11591627

[B2] Widlansky ME Gokce N Keaney JF Jr Vita JA The clinical implications of endothelial dysfunction J Am Coll Cardiol 2003 42 7 1149 1160 10.1016/S0735-1097(03)00994-X 14522472

[B3] Esper RJ Nordaby RA Vilarino JO Paragano A Cacharron JL Machado RA Endothelial dysfunction: a comprehensive appraisal Cardiovasc Diabetol 2006 5 4 10.1186/1475-2840-5-4 16504104 PMC1434727

[B4] Corretti MC Anderson TJ Benjamin EJ Celermajer D Charbonneau F Creager MA Deanfield J Drexler H Gerhard-Herman M Herrington D Guidelines for the ultrasound assessment of endothelial-dependent flow-mediated vasodilation of the brachial artery: a report of the international brachial artery reactivity task force J Am Coll Cardiol 2002 39 2 257 265 10.1016/S0735-1097(01)01746-6 11788217

[B5] Kuvin JT Mammen A Mooney P Alsheikh-Ali AA Karas RH Assessment of peripheral vascular endothelial function in the ambulatory setting Vasc Med 2007 12 1 13 16 10.1177/1358863X06076227 17451088

[B6] Celermajer DS Reliable endothelial function testing: at our fingertips? Circulation 2008 117 19 2428 2430 10.1161/CIRCULATIONAHA.108.775155 18474821

[B7] Selamet Tierney ES Newburger JW Gauvreau K Geva J Coogan E Colan SD de Ferranti SD Endothelial pulse amplitude testing: feasibility and reproducibility in adolescents J Pediatr 2009 154 6 901 905 10.1016/j.jpeds.2008.12.028 19217124

[B8] Nohria A Gerhard-Herman M Creager MA Hurley S Mitra D Ganz P Role of nitric oxide in the regulation of digital pulse volume amplitude in humans J Appl Physiol 2006 101 2 545 548 10.1152/japplphysiol.01285.2005 16614356

[B9] Bonetti PO Pumper GM Higano ST Holmes DR Jr Kuvin JT Lerman A Noninvasive identification of patients with early coronary atherosclerosis by assessment of digital reactive hyperemia J Am Coll Cardiol 2004 44 11 2137 2141 10.1016/j.jacc.2004.08.062 15582310

[B10] Kuvin JT Patel AR Sliney KA Pandian NG Sheffy J Schnall RP Karas RH Udelson JE Assessment of peripheral vascular endothelial function with finger arterial pulse wave amplitude Am Heart J 2003 146 1 168 174 10.1016/S0002-8703(03)00094-2 12851627

[B11] Hamburg NM Keyes MJ Larson MG Vasan RS Schnabel R Pryde MM Mitchell GF Sheffy J Vita JA Benjamin EJ Cross-sectional relations of digital vascular function to cardiovascular risk factors in the framingham heart study Circulation 2008 117 19 2467 2474 10.1161/CIRCULATIONAHA.107.748574 18458169 PMC2734141

[B12] Haller MJ Stein J Shuster J Theriaque D Silverstein J Schatz DA Earing MG Lerman A Mahmud FH Peripheral artery tonometry demonstrates altered endothelial function in children with type 1 diabetes Pediatr Diabetes 2007 8 4 193 198 10.1111/j.1399-5448.2007.00246.x 17659060

[B13] Truschel E Jarczok MN Fischer JE Terris DD High-throughput ambulatory assessment of digital reactive hyperemia: concurrent validity with known cardiovascular risk factors and potential confounding Prev Med 2009 49 6 468 472 10.1016/j.ypmed.2009.09.019 19804795

[B14] Fisher ND Hughes M Gerhard-Herman M Hollenberg NK Flavanol-rich cocoa induces nitric-oxide-dependent vasodilation in healthy humans J Hypertens 2003 21 12 2281 2286 10.1097/00004872-200312000-00016 14654748

[B15] Barringer TA Hatcher L Sasser HC Potential benefits on impairment of endothelial function after a high-fat meal of 4 weeks of flavonoid supplementation Evid Based Complement Alternat Med 2011 6 doi:10.1093/ecam/nen048. Article ID 796958 10.1093/ecam/nen048 PMC3137609 18955351

[B16] Bonetti PO Barsness GW Keelan PC Schnell TI Pumper GM Kuvin JT Schnall RP Holmes DR Higano ST Lerman A Enhanced external counterpulsation improves endothelial function in patients with symptomatic coronary artery disease J Am Coll Cardiol 2003 41 10 1761 1768 10.1016/S0735-1097(03)00329-2 12767662

[B17] Rubinshtein R Kuvin JT Soffler M Lennon RJ Lavi S Nelson RE Pumper GM Lerman LO Lerman A Assessment of endothelial function by non-invasive peripheral arterial tonometry predicts late cardiovascular adverse events Eur Heart J 2010 31 9 1142 1148 10.1093/eurheartj/ehq010 20181680

[B18] Hopsu L Simonen R Halonen J Konttinen J Mattila E Lindholm H Leino T Health promotion in occupational health care setting: effects of an intensive health promotion: Nuadu study Barents Newsl Occup Health Saf 2010 13 56 59

[B19] Tuomi K Ilmarinen J Jahkola A Katajarinne L Tulkki A Work Ability Index 2006 Helsinki, Finland: Finnish Institute of Occupational Health

[B20] Lindstrom J Tuomilehto J The diabetes risk score: a practical tool to predict type 2 diabetes risk Diabetes Care 2003 26 3 725 731 10.2337/diacare.26.3.725 12610029

[B21] 2008 Physical Activity Guidelines for Americans http://www.health.gov/PAGuidelines

[B22] Bush K Kivlahan DR McDonell MB Fihn SD Bradley KA The AUDIT alcohol consumption questions (AUDIT-C): an effective brief screening test for problem drinking. Ambulatory care quality improvement project (ACQUIP). alcohol Use disorders identification test Arch Intern Med 1998 158 16 1789 1795 10.1001/archinte.158.16.1789 9738608

[B23] Babor TF Higgins-Biddle JC Saunders JB Monteiro MG Alcohol use Disorders Identification Test—Guidelines for use in Primary Health Care 1990 2 Geneva, Switzerland: WHO

[B24] Friedewald WT Levy RI Fredrickson DS Estimation of the concentration of low-density lipoprotein cholesterol in plasma, without use of the preparative ultracentrifuge Clin Chem 1972 18 6 499 502 4337382

[B25] Miller VM Sex-based differences in vascular function Women’s Health (Lond Engl) 2010 6 5 737 752 10.2217/whe.10.53 20887171

[B26] The National FINRISK 2007 Study Publications of the National Public Health Institute 2008 Helsinki: National Public Health Institute

[B27] Libby P Inflammation in atherosclerosis Nature 2002 420 6917 868 874 10.1038/nature01323 12490960

[B28] Dhindsa M Sommerlad SM DeVan AE Barnes JN Sugawara J Ley O Tanaka H Interrelationships among noninvasive measures of postischemic macro- and microvascular reactivity J Appl Physiol 2008 105 2 427 432 10.1152/japplphysiol.90431.2008 18483158 PMC2519948

[B29] Jarvisalo MJ Jartti L Marniemi J Ronnemaa T Viikari JS Lehtimaki T Raitakari OT Determinants of short-term variation in arterial flow-mediated dilatation in healthy young men Clin Sci (Lond) 2006 110 4 475 482 10.1042/CS20050333 16396629

